# Anxiolytic, Promnesic, Anti-Acetylcholinesterase and Antioxidant Effects of Cotinine and 6-Hydroxy-L-Nicotine in Scopolamine-Induced Zebrafish (*Danio rerio*) Model of Alzheimer’s Disease

**DOI:** 10.3390/antiox10020212

**Published:** 2021-02-01

**Authors:** Razvan Stefan Boiangiu, Marius Mihasan, Dragos Lucian Gorgan, Bogdan Alexandru Stache, Lucian Hritcu

**Affiliations:** Department of Biology, Faculty of Biology, Alexandru Ioan Cuza University of Iasi, 700506 Iasi, Romania; marius.mihasan@uaic.ro (M.M.); lucian.gorgan@uaic.ro (D.L.G.); alexandru.stache@student.uaic.ro (B.A.S.)

**Keywords:** Alzheimer’s disease, nicotine, cotinine, 6-hydroxy-L-nicotine, scopolamine, memory, anxiety, acetylcholinesterase, oxidative stress, zebrafish

## Abstract

Cotinine (COT) and 6-hydroxy-L-nicotine (6HLN) are two nicotinic derivatives that possess cognitive-improving abilities and antioxidant properties in different rodent models of Alzheimer’s disease (AD), eluding the side-effects of nicotine (NIC), the parent molecule. In the current study, we evaluated the impact of COT and 6HLN on memory deterioration, anxiety, and oxidative stress in the scopolamine (SCOP)-induced zebrafish model of AD. For this, COT and 6HLN were acutely administered by immersion to zebrafish that were treated with SCOP before testing. The memory performances were assessed in Y-maze and object discrimination (NOR) tasks, while the anxiety-like behavior was evaluated in the novel tank diving test (NTT). The acetylcholinesterase (AChE) activity and oxidative stress were measured from brain samples. The RT-qPCR analysis was used to evaluate the *npy*, *egr1*, *bdnf*, and *nrf2a* gene expression. Our data indicated that both COT and 6HLN attenuated the SCOP-induced anxiety-like behavior and memory impairment and reduced the oxidative stress and AChE activity in the brain of zebrafish. Finally, RT-qPCR analysis indicated that COT and 6HLN increased the *npy*, *egr1*, *bdnf*, and *nrf2a* gene expression. Therefore, COT and 6HLN could be used as tools for improving AD conditions.

## 1. Introduction

Alzheimer’s disease (AD) is a progressive neurodegenerative disorder that affects over 50 million people worldwide, representing thus the most prevalent form of dementia and the fifth leading cause of death [1,2]. AD is associated with memory deficits and cognitive decline, although several neuropsychiatric symptoms, including apathy and anxiety, were also observed from the early stages of the disease [3,4]. AD neuropathology is characterized by intra- and extracellular accumulation of beta-amyloid (Aβ) peptide, the intracellular formation of neurofibrillary tangles (NFTs) of protein tau hyperphosphorylated, and the degeneration of cholinergic neurons accompanied by a reduction in acetylcholine (ACh) levels [5,6,7]. The cholinergic hypothesis of AD focuses on the degeneration of cholinergic neurons from the nucleus basalis of Meynert, which are involved in cognitive function, contributing thus to the memory loss exhibited by the AD patients [7,8]. The cholinergic transmission is achieved through nicotinic acetylcholine receptors (nAChRs) and muscarinic acetylcholine receptors (mAChRs), two families of ACh-binding receptors involved in cognitive processes and affected in AD [9].

Zebrafish (*Danio rerio*) is a small, hardy freshwater fish that originated from India, which was initially used as a model organism for studying vertebrate development [10]. The neuroanatomic and neurochemical pathways of the zebrafish brain display a great degree of similarity with those of the human brain. Moreover, the psychological, emotional, and social behavioral patterns are also very similar [10,11]. The zebrafish genome is fully sequenced and was shown to be evolutionarily conserved when compared to the human genome (~ 70% of similarity). The zebrafish has several gene orthologs similar to those mutated in familial AD [10,11,12,13]. Thereby, the zebrafish models have been successfully used to simulate AD pathology [11]. Scopolamine (SCOP), an antagonist of mAChRs, has been frequently used in experimental animals to produce learning and memory impairments, thus mimicking a type of dementia observed in AD [14].

Nicotine (NIC, (S)-3-(1-methylpyrrolidin-2-yl)pyridine), the alkaloid found in tobacco leaves, promotes the function of ACh by binding to nAChRs in the brain and enhancing performance in several domains of cognitive functions, including attention, working memory, and learning [15,16]. Additionally, NIC reduces oxidative stress [17], suppresses neuroinflammation [18], and prevents the Aβ aggregation in the brain [19]. Nevertheless, its therapeutic use in AD has been limited due to undesirable cardiovascular [20] and addictive [21] side effects as well as the bad publicity associated with smoking [22]. Considering the positive effects on the central nervous system (CNS), NIC provides a strong scaffold for developing new AD therapeutic agents in the form of nicotinic derivatives that eludes the side effects of NIC [23].

In mammals, approximately 80–85% of NIC is metabolized into cotinine (COT, (S)-1-methyl-5-(pyridin-3-yl)pyrrolidin-2-one) by the liver enzymes, such as cytochrome P450 2A6 (CYP2A6) [24]. Compared to NIC, COT is less efficient in crossing the blood-brain barrier, but has a good safety profile, no addictive, cardiovascular, or behavioral effects in humans, has a longer half-life time (19–20 h vs. 2 h), and much lower toxicity [24,25]. The difference between the half-life time of COT and NIC might suggest that COT could underlie the NIC’s prolonged effects in CNS [26].

6-Hydroxy-L-nicotine (6HLN, 5-[(2S)-1-methylpyrrolidin-2-yl]pyridin-2-ol) is the first metabolic intermediate found in the NIC catabolic pathway encoded by the pAO1 megaplasmid of *Paenarthrobacter nicotinovorans*, a soil Gram-positive bacteria [27,28]. The degradation begins with the hydroxylation of the pyridine ring of NIC by the nicotine-dehydrogenase (NDH) enzyme, resulting in 6HLN. During NIC consumption, 6HLN is accumulating in the media for a short period [29] after which the pyrrolidine ring is further oxidized by the 6-hydroxy-L-nicotine oxidase (6HLNO), resulting in 6-hydroxy-methylmyosmine [28,30].

**Figure 1:**
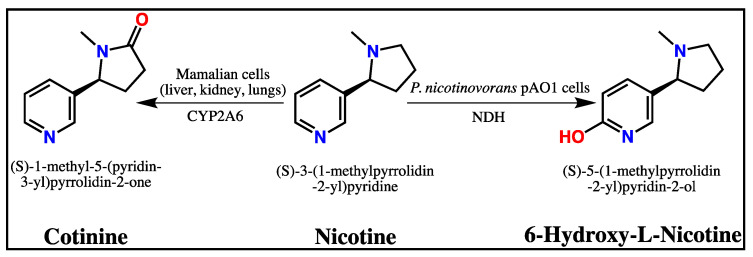
The comparison of the chemical structures of nicotine and nicotinic derivatives: cotinine and 6-hydroxy-L-nicotine (CYP2A6—Cytochrome P450 2A6; NDH—nicotine-dehydrogenase).

We have previously shown that 6HLN attenuates the cognitive deficits and recovered the antioxidant capacity in the hippocampus of several rodent models of AD [31,32,33]. Furthermore, it was demonstrated that COT ameliorates the memory impairments and decrease the Aβ load in AD mice [34,35]. The purpose of the current study was to investigate the effects of two structural related NIC metabolites (Figure 1), COT and 6HLN, on anxiety-like behavior, memory deficits, and oxidative stress in a zebrafish model of AD induced by SCOP. Also, we evaluated the effects of these compounds on *bdnf*, *npy*, *egr1*, and *nrf2a* gene expression. To our knowledge, this is the first study that examines the effects of COT and 6HLN on zebrafish.

## 2. Materials and Methods

### 2.1. Animals and Housing

A total number of 100 adult (6–8 months) zebrafish (*Danio rerio*), wild type short-fin strain from both sexes (50:50), was purchased from an authorized company (Pet product S.R.L., Bucharest, Romania). The fish were housed for two weeks in three tanks of 70 L each, constantly aerated and illuminated for 14 h starting from 8 a.m. The water temperature was kept at 27 ± 0.5 °C, pH 7 ± 0.15, dissolved oxygen 6 ± 0.1 mg/L, total ammonia < 0.01 mg/L, total hardness 6 mg/L, and alkalinity of 22 mg/L CaCO_3_. The water parameters were not changed between the treatments. The fish were fed twice per day with commercial food NovoMalawi (JBL, Neuhofen, Germany). This study was previously approved by the local Ethics Committee for animal experimentation (No. 02/30.06.2020) and was conducted following the Directive 2010/63/EU of the European Parliament and of the Council of 22 September 2010 on the protection of animals in scientific purposes. Efforts were made to reduce the number of animals utilized and their suffering.

### 2.2. Chemicals and Reagents

Nicotine (NIC, CAS No. 54-11-5), cotinine (COT, CAS No. 486-56-6), galantamine hydrobromide (GAL, CAS No. 1953-04-4), imipramine hydrochloride (IMP, CAS No. 113-52-0), and scopolamine hydrobromide (SCOP, CAS No. 114-49-8) of highest purity were purchased from a well-known supplier (Sigma Aldrich, Darmstadt, Germany). 6-Hydroxy-L-nicotine (6HLN) was chemically synthesized and was provided by Prof. dr. Roderich Brandsch (Albert Ludwigs University of Freiburg, Freiburg, Germany). All the chemical used was obtained from Sigma-Aldrich, Darmstadt, Germany.

### 2.3. Treatment and Group Division

**Figure 2:**
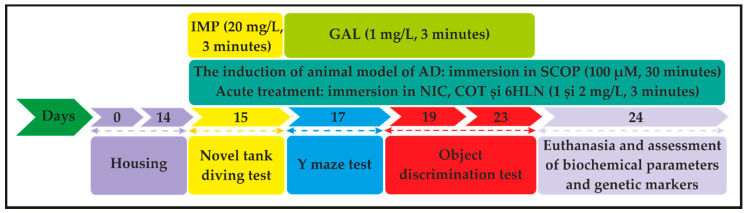
The timeline and experimental design of the study (IMP-imipramine; GAL-galantamine; AD-Alzheimer’s disease; SCOP-scopolamine; NIC-nicotine; COT-cotinine; 6HLN-6-hydroxy-L-nicotine).

Stock solutions of 1 mg/mL of NIC, COT, 6HLN, and GAL and 20 mg/mL of IMP were prepared freshly in distilled water. SCOP solution 100 µM was prepared in 2 L of distilled water and, to minimize the variables that could affect behavior, it was prepared before use and changed after each exposure. The animals were assigned into ten experimental groups (10 fish per group), each group being housed in 24 L tanks as follow: the control group, the SCOP group, the GAL group (used as a positive control in memory tasks), the IMP group (used as a positive control in anxiety test), two NIC groups (1 and 2 mg/L), two COT groups (1 and 2 mg/L) and two 6HLN groups (1 and 2 mg/L). Before performing a behavioral task, fish (except those from the control group) were individually placed in SCOP solution 100 µM for 30 min to induce the zebrafish model of AD, as previously described [36,37,38]. Subsequently, fish were individually subjected to a 3 min acute treatment with 1 and 2 mg/L of NIC, COT, or 6HLN in a 0.5 L pre-treatment beaker (Figure 2). Before being placed in the testing tank, the fish were allowed to rest for 5 min in their home tanks. The doses of 1 and 2 mg/L of NIC, COT, and 6HLN and the time of exposure were chosen based on previous reports [39,40,41,42]. Similarly, IMP and GAL were acutely administered in doses of 20 mg/L and 1 mg/L, respectively.

### 2.4. Behavioral Tasks

The zebrafish swimming behavior within the in vivo tasks was recorded with a Logitech C922 Pro HD Stream webcam (Logitech, Lausanne, Switzerland), and the recordings were analyzed using ANY-maze software v6.3 (Stoelting Co., Wood Dale, IL, USA).

#### 2.4.1. Novel Tank Diving Test

In the novel tank diving test (NTT), the zebrafish exhibits robust behavioral responses to novelty-provoked anxiety. The NTT protocol applied in this study was described before by Cachat et al. [43] and Rosemberg et al. [44]. The testing apparatus consisted of a trapezoidal glass tank filled with 1.5 L of home tank water and having the following dimensions: 23.9 cm along the bottom × 28.9 cm at the top × 15.1 cm high with 15.9 cm along the diagonal side, 7.4 cm wide at the top and 6.1 cm wide at the bottom. The fish were individually placed in the testing tank and their behavior was recorded for 6 min with a webcam placed at 40 cm in the front of the tank. The tank was virtually divided into the top zone and bottom zone, respectively. To measure anxiety-like behavior and the locomotor activity of the zebrafish, we used the behavioral endpoints described previously by Cachat et al. [43].

#### 2.4.2. Y-Maze Test

The zebrafish memory and response to novelty were investigated using a protocol of the Y-maze task that was formerly described by Cognato et al. [45] and Zanandrea et al. [46]. The fish were tested in a Y-shaped glass aquarium, having three arms in size of 25 × 8 × 15 cm (L × l × h) and filled with 3 L of home tank water. Different recognizable geometric shapes, such as triangles, circles, and squares, were placed on the outer walls of each arm. The arms of the maze were set randomly as follows: (i) the start arm (A) from which the fish begins the test, (ii) the other arm (B) that is permanently open and (iii) the novel arm (C) which is blocked during the training period and open in the testing phase. The center of the Y-maze was not taken into account for the analysis. This task was performed in two stages separated by 1 h between them in order to assess the response to novelty and the spatial recognition memory. During the first stage (training session), the fish was allowed to explore the start and the other arm for 5 min, while the novel arm was kept closed. In the second stage (testing session) the fish was placed in the start arm and was allowed to explore the entire maze for 5 min. The time spent in the novel arm (% of total time), total distance travelled (m), and the turn angle (°) were the behavioral endpoints examined in this task.

#### 2.4.3. Object Discrimination Test

Object discrimination task, also known as the novel object recognition (NOR) task, was described before by Stefanello et al. [47] and Gaspary et al. [48] and used in the current study to assess the ability of the animals to recognize a new object in the environment. This behavioral test was performed in a ~20 L glass aquarium in size of 30 × 30 × 30 cm (L × l × h) and filled with 6 cm of home tank water. To avoid reflection and external interference, the outer floor and walls were covered with black plastic. In the habituation session, the fish was placed in the testing tank without the objects for 5 min, twice per day (5 h period between trials), and for three successive days. On the fourth day, the animals were exposed for 10 min to two identical objects (two red cubes) in the training session. Post-training, the fish were submitted to a 1 h-retention interval in which the animal model of AD was induced, and the treatment was acutely administered. In the testing session, one of the familiar objects (F, red cube) was replaced with a novel object (N, green cube), and the fish was allowed to explore both objects for 10 min. The preference percentage was the behavioral parameter examined in the testing phase, and it was calculated using the formula: [exploration time of N/(exploration time of F + exploration time of N)] × 100. Preference scores higher than 50% (chance marked with a dashed line in the graph) indicates a relative preference for N, below 50% indicates a relative aversion for N and a 50% score indicates no preference for N compared to F [49]. The exploration area was defined as increasing once the size of the object area [50]. and thus, we considered the exploration activity when the fish was at a distance of up to 2.5 cm from each side of the cube.

### 2.5. Biochemical Parameter Analysis

Shortly after performing the in vivo tasks, the fish were euthanized by rapid cooling, as this method does not cause biochemical or physiological changes that could prevent post-mortem analysis [51]. Subsequently, the fish were dissected using the procedure described by Gupta and Mullins [52], and whole brains were collected for biochemical analysis. A pool of two brains was considered one independent sample. The brain tissues were gently homogenized (1:10 ratio, *w*/*v*) for 1 min at 1000 rpm in cold 0.1 M potassium phosphate buffer (pH 7.4), 1.15% KCl with the Mikro-Dismembrator U mill (Sartorius, New York, NY, USA) equipped with 3 mm diameter magnetic balls (Sartorius Stedim Biotech GmbH, Goettingen, Germany). The homogenate was centrifuged for 15 min at 14,000 rpm and the supernatant was used to determine the total content of soluble protein, to assess the superoxide dismutase (SOD), catalase (CAT), glutathione peroxidase (GPX), and acetylcholinesterase- (AChE)-specific activities and to estimate the level of reduced glutathione (GSH), malondialdehyde (MDA) and carbonylated proteins.

#### 2.5.1. Protein Concentration Determination

The soluble protein concentration was estimated using the Bicinchoninic Acid (BCA) Kit (Sigma Aldrich, Darmstadt, Germany), which was developed based on the method of Smith et al. [53].

#### 2.5.2. Evaluation of AChE Activity

The AChE activity was monitored from the brain samples using the colorimetric method of Ellman et al. [54]. A total volume of 600 µL reaction mixture was prepared by adding sodium phosphate buffer solution 0.25 M (pH 7.4), 5,5′-dithiobis-2-nitrobenzoic acid solution (DTNB) 1 mM, enzymatic extract, and acetylthiocholine (ATCh) chloride 5 mM. The reaction was stopped after 10 min with acetone, and subsequently, the formation of the yellow color corresponding to 2-nitro-5-thiobenzoate anion was monitored at 412 nm. The AChE activity was expressed as nmoles of ATCh hydrolyzed/min/mg protein.

#### 2.5.3. Evaluation of SOD Activity

The SOD activity was measured based on the protocol previously described by Winterbourn et al. [55]. Briefly, we monitored the ability of the enzyme to inhibit the reduction of Nitro Blue Tetrazolium salt (NBT) by the superoxide free radicals generated by the photoreduction of riboflavin. For this, we prepared a 1.4 mL reaction mixture containing potassium phosphate buffer 0.067 M (pH 7.8), EDTA 0.1 M (pH 7.8), enzymatic extract, NBT 1.5 mM and riboflavin 0.12 mM. The reaction volume was exposed 30 min to light, and the resulting blue formazan was followed at 560 nm. One SOD unit represented the amount of enzyme capable of inhibiting the NBT reduction rate by 50%. The enzyme activity was expressed as SOD units/mg protein.

#### 2.5.4. Evaluation of CAT Activity

The CAT activity was measured using the colorimetric method of Sinha [56]. Briefly, 125 µL enzymatic extract reacted with an equal volume of H_2_O_2_ 0.16 M for 3 min at 37 °C. The reaction was stopped with 500 µL potassium dichromate: acetic acid reagent, and the mixture was incubated at 95 °C for 15 min. The green color corresponding to chromic acetate was read at 570 nm. One CAT unit represented 1 µmol of H_2_O_2_ consumed in 3 min. The enzyme activity was expressed as CAT units/mg protein.

#### 2.5.5. Evaluation of GPX Activity

The GPX activity was measured using the protocol described by Fukuzawa and Tokumura [57]. A total volume of 650 µL reaction mixture containing 78 µL enzymatic extract, 475 µL of sodium phosphate buffer 0.25 M (pH 7.4), 36 µL of EDTA 25 mM, and 36 µL NaN_3_ 0.4 M was incubated 10 min at 37 °C. The reaction was initiated by adding 50 µL of GSH 50 mM and 36 µL of H_2_O_2_ 50 mM and the tubes were incubated again at 37 °C for 10 min. The reaction was stopped with 730 µL metaphosphoric acid 7% and the tubes were centrifuged 10 min at 14,000 rpm. A volume of 100 µL supernatant was mixed with 1270 µL disodium phosphate solution 0.3 M and 136 µL DTNB 0.04%. The remaining GSH reacted with DTNB, and the resulting yellow product was followed at 412 nm. One GPX unit represented the amount of enzyme necessary to oxidize 1 µmol GSH per minute. The enzyme activity was expressed as GPX units/mg protein.

#### 2.5.6. GSH Content

To determine the total content of GSH from brain samples, we applied the procedure developed by Anderson [58] and modified by Salbitani et al. [59]. A volume of 200 µL supernatant was mixed with 1100 µL disodium phosphate 0.3 M and 130 µL DTNB 0.04%. The mixture was incubated for 2 min at room temperature, and the yellow product was followed at 412 nm. The amount was expressed as µg GSH/mg protein.

#### 2.5.7. Carbonylated Proteins Levels

The content of carbonylated proteins was examined using the method developed by Oliver et al. [60] and modified by Luo and Wehr [61]. This method is based on the reaction between the carbonyl groups and 2,4-dinitrophenylhydrazine (DNPH), resulting in the protein-bound 2,4-dinitrophenylhydrazones. An amount of 1 mg protein was precipitated with trichloroacetic acid (TCA, *w*/*v*) 20% and centrifuged at 14,000 rpm for 5 min. The supernatant was discarded, and the pellet was dissolved in DNPH 0.2% (prepared in HCl 2 N) and precipitated again with TCA 20%. The tubes were centrifuged for 5 min at 14,000 rpm, and the pellet was washed three times with 1 mL ethanol-ethyl acetate (1:1, *v*/*v*). The samples were left to dry at room temperature for 10 min, and the pellet was solubilized in guanidine hydrochloride 6 M (prepared in monopotassium phosphate 20 mM). The extinctions were read at 370 nm, and the amount of carbonylated proteins was expressed as nmoles DNPH/mg protein.

#### 2.5.8. MDA Levels

The MDA content in the brain was assessed using a High-Performance Liquid Chromatography (HPLC) method that is based on 2-thiobarbituric acid (TBA) assay and which was formerly described by Domijan et al. [62] and modified later by Vaides-Negustor and Mihasan [63]. Briefly, 50 µL samples or standards were mixed with 12.5 µL NaOH 3 M and incubated at 60 °C for 30 min in stirring conditions (300 rpm). Subsequently, 500 µL H_2_SO_4_ 0.5 M and 250 µL TCA 20% were added to the mixture and the tubes were centrifuged 10 min at 3000 rpm. A volume of 500 µL supernatant was mixed with 250 µL TBA 0.355% and the tubes were incubated at 90 °C for 30 min and then centrifuged 30 min at 13,000 rpm. HPLC analysis started by injecting 20 µL of sample or standard in a Shimadzu Prominence system (Shimadzu Corporation, Kyoto, Japan) equipped with SIL-20AC autosampler, two LC-20AD pumps, SPD M20A DAD detector, CTO-20AC column oven, and a Zorbax Eclipse XDB–C18 reverse-phase (RP) column (Agilent Technologies, Santa Clara, CA, USA) with a length of 250 mm and 3 µm particle size. The mobile phase consisted of a mixture of methanol (Carl Roth, Karlsruhe, Germany) and monopotassium phosphate 30 mM pH 6.7 (35:65 ratio) and the samples or standards were run for 20 min using a flow rate of 1 mL/min. Different concentrations of 1,1,3,3-tetraethoxypropane (TEP, Sigma-Aldrich, Darmstadt, Germany) were used to create the standard curve. The pink adducts corresponding to TEP- or MDA-TBA complex were followed at 532 nm and the elution took place at 9.5 ± 0.2 min after injection. The final calculation was based on the height or area of the peaks, and the results were expressed in µmoles MDA/L.

### 2.6. RNA Purification and Real-Time Quantitative PCR (RT-qPCR) Analysis

The *npy*, *bdnf*, *egr1*, and *nrf2a* genes expression in the zebrafish brain was investigated using an RT-qPCR procedure as formerly described by Ionita et al. [64] and Boiangiu et al. [33]. For this, total RNA was purified from ~5 mg brain tissue using the Maxwell^®^ 16 Tissue Lev Total RNA Purification kit (Promega, Madison, WI, USA) according to the manufacturer instructions and the automatic purification system Maxwell^®^ 16 Instrument AS2000 (Promega, Madison, WI, USA). Both reverse transcription and RT-qPCR analysis were conducted in a single-step amplification reaction using GoTaq^®^ 1-Step RT-qPCR System (Promega, Madison, WI, USA) on a 5-plex HRM Rotor-Gene 6000 (Corbett, CA, USA) rotary real-time PCR machine. The amplification reaction took place in 10 µL volume and contained the following reagents: GoTaq^®^ Probe, qPCR Master Mix 2X (Promega, Madison, WI, USA), GoScript™ RT Mix for 1-Step RT-qPCR 50X, 300 nm pre-design primers for *Danio rerio* (Table 1), 100 ng total RNA template and Nuclease-free water up to volume. The results were analyzed using Rotor-Gene Q-Pure Detection Software v2.2.3 (Qiagen, Redwood City, CA, USA).

### 2.7. Statistic Interpretation

All data expressed as means ± standard error of the mean (S.E.M.), were statistically analyzed by one-way analysis of variance (ANOVA) followed by Tukey’s *post hoc* multiple comparison tests using GraphPad Prism v8.3 software (La Jolla, CA, USA). A statistically significant difference was considered for a *p* < 0.05. The Pearson correlation coefficient (r) was also used to correlate several behavioral or biochemical parameters with MDA, the product of lipid peroxidation, or *nrf2a* and *egr1*, markers of gene expression.

## 3. Results and Discussion

### 3.1. The Effects of Cotinine and 6-Hydroxy-L-Nicotine on Anxiety

Neuropsychiatric symptoms, such as apathy and anxiety, are highly prevalent in AD patients and were associated with Aβ deposition and cognitive decline, indicating that these symptoms are early clinical manifestations of AD [4,65]. Accumulating evidence has suggested that different neurotransmitter systems, including cholinergic neurotransmission, modulate anxiety-like behaviors [40,66]. In this study, we evaluated the effects of COT and 6HLN acute administration on anxiety-like behavior in the SCOP-induced zebrafish model of AD.

**Figure 3:**
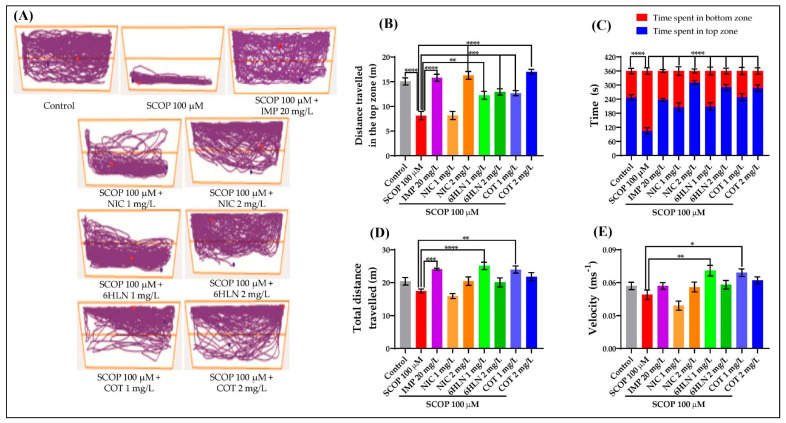
The effects of nicotine (NIC), 6-hydroxy-L-nicotine (6HLN), and cotinine (COT) (1 and 2 mg/L) administration in scopolamine (SCOP)-treated zebrafish on anxiety-like behavior and locomotor activity evaluated within the novel tank diving test (NTT). (**A**) The zebrafish locomotion tracking pattern in the NTT according to the experimental groups they belong to. The blue dot represents the beginning of the route while the red dot represents the end of the route. The distance travelled in the top (**B**) and the time spent in the top/bottom (**C**) were endpoints for measuring the anxiety, whereas the total distance travelled (**D**) and the velocity (**E**) were endpoints for measuring the locomotor activity. The values are expressed as means ± S.E.M. (*n* = 10). ANOVA analysis identified overall significant differences between the experimental groups for (**B**) F(8,81) = 21.35, *p* < 0.0001, (**C**) F(8,162) = 42.29, *p* < 0.0001, (**D**) F(8,81) = 8.555, *p* < 0.0001 and (**E**) F(8,81) = 6.172, *p* < 0.0001. For Tukey *posthoc* analyses—**** *p* < 0.0001, *** *p* < 0.001, ** *p* < 0.01 and * *p* < 0.05.

NTT was used to assess the natural neophobic response of the zebrafish, expressed as reduced exploration, increased freezing, and/or unorganized irregular locomotion. Instead, a reduction of anxiety is accompanied by an increase of exploration with a low number of freezing bouts and erratic movements [67]. According to the representative tracking plots depicted in Figure 3A, we noticed a different swimming pattern of the zebrafish corresponding to their treatment, especially of those treated with SCOP, which displayed intense activity in the lower half of the tank, thus suggesting an anxiogenic profile. The zebrafish that belonged to the control group initially showed exploratory activity in the bottom zone of the tank followed by a general migration during the test. Moreover, we also noticed intense exploratory behavior among the fish treated with 6HLN and COT in the upper half of the tank, especially when the dose of 2 mg/L was used (Figure 3A). The effects of COT and 6HLN acute treatment on anxiety-like behavior in zebrafish exposed to SCOP (100 µM) were assessed in NTT by measuring the distance travelled and time spent by fish in the top zone of the tank. SCOP administration elicited a robust anxiogenic response, causing a significant decrease in the distance travelled (*p* < 0.0001, Figure 3B) and a significant reduction of the time spent in the top zone (*p* < 0.0001, Figure 3C) compared to the control and IMP + SCOP groups. IMP is a tricyclic antidepressant that appears to be also effective for reducing overall anxiety [68], and for this reason, it was used as a positive control within the NTT task. The acute administration of both doses of 1 and 2 mg/L of COT and 6HLN ameliorated the anxiogenic effect of SCOP by increasing the distance travelled (Figure 3B) by the fish in the top zone concomitant with the increase of the time spent by the fish in this area (Figure 3C). Also, the same anxiolytic profile of the COT and 6HLN was observed in other parameters measured in NTT (Supplementary Figure S1). Analyzing the endpoints corresponding to the locomotor activity, we found no significant difference between the SCOP-treated group and the control group, whereas the fish travelled similar distances (Figure 3D) with the same velocity (Figure 3E). Nevertheless, the 1 mg/L dose of COT and 6HLN administered to SCOP-treated zebrafish was able to enhance the locomotor activity by significantly increasing the total distance travelled (*p* < 0.01 for COT and *p* < 0.0001 for 6HLN, Figure 3D) and the swimming speed (*p* < 0.05 for COT and *p* < 0.01 for 6HLN, Figure 3E). This increase in the locomotor parameters might suggest hyperactivity.

Our results indicated that COT and 6HLN treatment reduced the anxiety-like behavior induced by SCOP in zebrafish. As a hallucinogen, SCOP exerts complex dose-dependent effects on CNS and both dose and exposure time may be important factors [69]. Hamilton et al. [70] chose a 30 min pre-treatment with SCOP (800 µM) as a behaviorally active dose, while other studies used a 30–60 min exposure with SCOP (100 or 200 µM) [37,38,71,72]. In the study performed by Volgin et al. [69], the zebrafish were exposed for 20 min to a pre-treatment with different concentrations of SCOP (60–240 mg/L), but the intermediary dose of 120 mg/L (~400 µM) was chosen as the biologically active dose. Thus, although SCOP dose and time of exposure appear to be comparable between the studies performed on zebrafish, they provide conflicting results, varying from lack of effects [71,72] to anxiolytic [70], pro-anxiogenic (which inhibits anxiolysis) [72], and anxiogenic responses [37,38,69,72,73]. In the current study, the pre-treatment with SCOP (100 µM) for 30 min elicited a robust anxiogenic response in the zebrafish without reducing their movement. NIC influences a large number of physiological processes in zebrafish, such as locomotion and anxiety [40,41,66]. By using experimental models, a dual role of NIC has been demonstrated, which promotes anxiolytic or anxiogenic effects due to acute or chronic exposure, respectively [39,40,42]. Duarte et al. [40] showed that acute administration of NIC (1 mg/L, for 3 min) prevents behavioral responses similar to anxiety in zebrafish. Moreover, Singer et. al. [39] demonstrated that NIC affects zebrafish behavior, regardless of their sex, in a manner that indicates an anxiety reduction, intensifying the swimming speed. In the experiment performed by Cachat et al. [67], acute administration of NIC (10 mg/L) to zebrafish displayed an anxiolytic effect in NTT. Acute exposure to NIC ditartrate (100 mg/L by immersion, for 3 min), with 5 min before testing, significantly reduced the time spent by the zebrafish at the bottom of the tank [73]. This effect was blocked by the co-administration of mecamylamine (non-selective and non-competitive antagonist of nAChRs), methylicaconitine (antagonist of α7 nAChRs), and dihydro-β-erythroidine (antagonist of α4β2 nAChRs), thus suggesting that the anxiolytic effects of NIC involved a direct response related to the binding of α4β2 and α7 nAChRs [73,74]. Similarly, due to structural resemblance with NIC, the anxiolytic properties of COT and 6HLN showed in this study could be associated with the binding to α4β2 and α7 nAChRs. In a recent study, we showed, using in silico tools, that these nicotinic derivatives might bind to α4β2 and α7 nAChRs with similar or higher affinity than NIC [33]. The COT effects on anxiety-like behavior were investigated in a mouse model of post-traumatic stress disorder (PTSD) induced by fear conditioning [75]. It has been shown that COT treatment reduced the anxiety after fear conditioning, and the post-treatment enhanced the extinction of contextual fear memory [75]. Moreover, Ionita et al. [76] recently investigated, using the elevated plus maze task, the anxiolytic effect of 6HLN compared to that of NIC in a rat model of AD induced by chlorisondamine (CHL). They observed that both NIC and 6HLN act as anxiolytic agents in this animal model, reducing the anxiety caused by CHL. Consistent with these studies, we showed that COT and 6HLN reduce the SCOP-induced anxiety-like behavior in zebrafish.

### 3.2. The Effects of Cotinine and 6-Hydroxy-L-Nicotine on Cognition

The cognitive deficits associated with AD could be modeled using pharmacological interventions. The cholinergic system, which mediates the learning and memory processes, is affected in AD. Thereby, the AD patients display reduced nicotinic and muscarinic binding sites, as well as reduced AChE activity [77]. SCOP, the antagonist of mAChRs, can produce amnesia in zebrafish, preserving the normal locomotor activity, thus suggesting that the cholinergic system of the fish is involved in learning and memory processes [77]. In this experiment, we used Y-maze and object discrimination tasks to evaluate the cognitive effects of 6HLN and COT in a zebrafish model of AD induced by immersion in SCOP.

**Figure 4:**
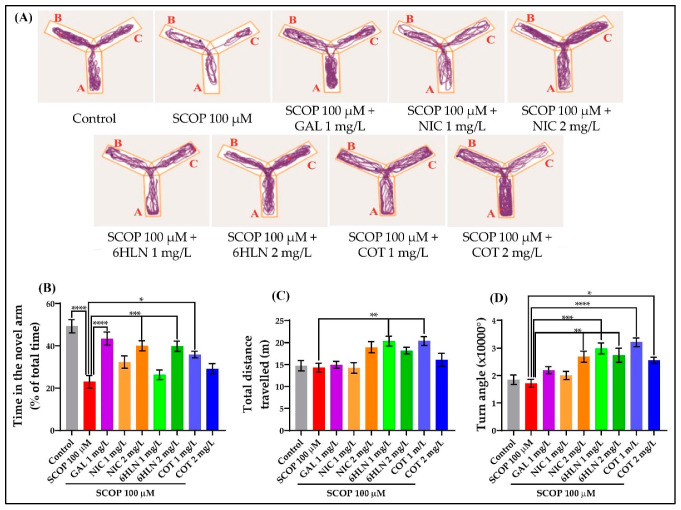
The effects of nicotine (NIC), 6-hydroxy-L-nicotine (6HLN), and cotinine (COT) (1 and 2 mg/L) administration in scopolamine (SCOP)-treated zebrafish on spatial memory and locomotor activity evaluated within the Y-maze task. (**A**) The representative locomotion tracking pattern of the zebrafish in the second stage of the Y-maze task according to the experimental groups they belong to. The arms of the maze were denoted with A (start arm), B (other arm), and C (novel arm). The blue dot represents the beginning of the route, while the red dot represents the end of the route. The time in the novel arm (**B**) represents a parameter for spatial memory, whereas the (**C**) total distance travelled and the (**D**) turn angle is measures of locomotor activity. The values are expressed as means ± S.E.M. (*n* = 10). ANOVA analysis identified overall significant differences between the experimental groups for (**B**) F(8,81) = 10.54, *p* < 0.0001, (**C**) F(8,81) = 5.342, *p* < 0.0001 and (**D**) F(8,81) = 8.96, *p* < 0.0001. For Tukey *posthoc* analyses—**** *p* < 0.0001, *** *p* < 0.001, ** *p* < 0.01and * *p* < 0.05.

The Y-maze test was applied to evaluate the response to novelty and the spatial memory of the zebrafish. As depicted in Figure 4A, the representatives tracking plots obtained after performing the Y-maze task revealed different swimming patterns according to the experimental groups. Although all fish explored the three arms of the maze, the animals that belonged to the group treated with SCOP (100 µM) showed reduced activity in the novel arm. However, administration of GAL, NIC, 6HLN, and COT increased the activity of the zebrafish in the arms of the Y-maze, especially in the novel arm. The spatial memory was assessed using as a parameter the time spent by the fish in the novel arm of the maze reported to the total exploration time. Figure 4B shows that the zebrafish treated with SCOP 100 µM spent significantly (*p* < 0.0001) less time in the novel arm than the animals from the control group or GAL + SCOP group, thus suggesting an amnesic effect of SCOP. GAL, a competitive, selective, and reversible AChE inhibitor, is used in AD therapy to improve cognitive function [78,79], and it was considered a positive control in the cognitive tasks performed in this study. 6HLN acute administration was able to counteract the memory deficits caused by SCOP treatment only when the 2 mg/L dose was used (*p* < 0.001, Figure 4B). Regarding COT, only the 1 mg/L dose ameliorated the SCOP-induced memory impairments in zebrafish (*p* < 0.05, Figure 4B). Although a slight increase in the time spent in the novel arm could be observed in the group treated with 2 mg/L of COT, no significant difference was detected. Given that any change in locomotor activity could influence the results of the Y-maze test, the exploratory behavior was assessed after SCOP administration and acute treatment with COT and 6HLN by quantifying the total distance travelled and the absolute turn angle. According to Tukey *posthoc* analyses, no significant differences in the locomotor activity were identified between the fish treated with SCOP (100 µM) and those belonging to the control group. However, similar to the results obtained in NTT, we noticed a significant increase (*p* < 0.01) in the total distance travelled by the fish co-treated with SCOP (100 µM) and 1 mg/L of 6HLN or COT (Figure 4C). Additionally, both doses of 6HLN and COT, but especially the one of 1 mg/L, also increased the absolute turn angle of the fish, thus supporting an enhancing effect of the treatment on the locomotor behavior of the zebrafish. Hence, COT and 6HLN alleviated the SCOP-induced spatial memory deficits in zebrafish within the Y-maze task.

**Figure 5:**
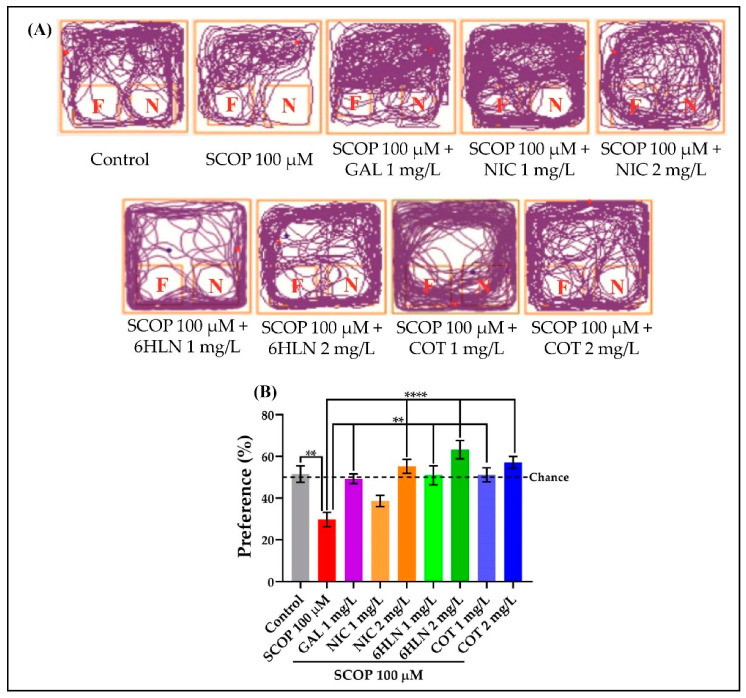
The effects of nicotine (NIC), 6-hydroxy-L-nicotine (6HLN), and cotinine (COT) (1 and 2 mg/L) administration in scopolamine (SCOP)-treated zebrafish on recognition memory evaluated within object discrimination task. (**A**) The locomotor tracking pattern of the zebrafish within the testing session of the object discrimination task according to the groups they belong to. The blue dot represents the beginning of the route, while the red dot represents the end of the route. The preference percentage (**B**) was considered the endpoint for recognition memory. The familiar and the novel objects were noted with F and N, respectively. The black dashed line (chance) indicates a 50% preference. The values are expressed as means ± S.E.M. (*n* = 10). ANOVA analysis revealed overall significant differences between groups for (**B**) F(8,81) = 8.078, *p* < 0.0001. For Tukey *posthoc* analyses—**** *p* < 0.0001, and ** *p* < 0.001.

In this experiment, the object discrimination test was used to evaluate the recognition memory of the zebrafish. These vertebrates are capable of discriminating between a familiar and a novel object [80] as well as recognize 3D geometric shapes [81]. Following the object discrimination test, we obtained the representative tracking plots showing the route travelled by the fish in the testing session according to experimental groups. As it is depicted in Figure 5A, the animals treated with SCOP 100 µM spent more time exploring the familiar object and less time exploring the novel object compared to animals from other groups. Acute treatment with GAL, NIC, 6HLN, and COT increased the exploration time of the novel object. Moreover, the fish from all the experimental groups initially displayed thigmotaxis behavior, an animal’s preference for staying near to the edge/side, avoiding the central open areas. However, this behavior was gradually reduced during the testing session. The recognition memory was expressed in percentages of preference and reflected the animal’s predilection for the novel object. Our results showed that treatment with SCOP 100 µM significantly decreased (*p* < 0.01) the preference of the zebrafish, compared with the animals from control and GAL + SCOP groups (Figure 5B), leading to scores lower than 50% and suggesting a relative aversion to the novel object. As in the Y-maze test, GAL was used as a positive control. According to Figure 5B, both doses of COT and 6HLN, but especially the 2 mg/L dose, reversed the recognition memory deficits caused by SCOP administration by significantly (*p* < 0.01and *p* < 0.0001 for 1 and 2 mg/L. respectively, Figure 5B) increasing the preference of the zebrafish for the novel object. The 2 mg/L dose of COT and 6HLN determined performances that exceeded the chance level (50%), thus indicating a relative preference to the novel object. Therefore, the nicotinic derivatives improve the recognition memory in the zebrafish model of AD induced by SCOP within the object discrimination task.

The results obtained in Y-maze and object discrimination tasks suggested a promnesic effect of COT and 6HLN in the zebrafish model of AD induced by immersion in SCOP. The cholinergic system is involved in many physiological processes, including synaptic plasticity, learning, and memory [82]. Cholinergic agonists can facilitate memory, while cholinergic antagonists can impair it [83]. SCOP is a mAChRs antagonist and causes amnesia in experimental models of dementia [14,47]. According to Cognato et al. [45], the zebrafish display a preference for the unexplored arm of the Y-maze. However, the pre-training SCOP treatment (50–200 µM) induced memory deficits within this task without causing locomotor deficits [45,46]. Moreover, Stefanello et al. [47] showed that SCOP administration (200 µM for 1 h) to zebrafish reduced the time spent by the fish in novel object area and abolished the interaction-like phenotypes in the object discrimination task.

It has been demonstrated that acute NIC exposure improves discrimination and memory function in zebrafish followed a 20–40 min latency, but not immediately after exposure [84,85,86]. This effect was reduced by mecamylamine administration immediately after performing the cognitive tasks but not when it was co-administered with NIC 40 min before testing. These findings led to the hypothesis that NIC enhances cognition through desensitizing and resensitizing nAChRs with increased response to endogenous ACh [85]. Also, this enhanced cognitive function was correlated with elevated levels of dihydroxyphenylacetic acid, the primary dopamine metabolite created during the synaptic reuptake of this neurotransmitter. This observation suggests that potentiated release of dopamine following NIC administration is associated with cognitive improvements [86]. The cognitive enhancement induced by NIC and its attenuation by the pre-treatment with mecamylamine in zebrafish were also observed in a memory study using the place preference test [87]. Similar results were also obtained by Braida et al. [88], which showed that NIC-induced cognitive enhancement in zebrafish within the T-maze task was reduced by using different nAChRs and mAChRs antagonists, supporting thus the involvement of the cholinergic system in the positive effects of NIC on memory. According to May et al. [80], the NIC tartrate administration (50 mg/L for 3 min) enhanced zebrafish familiarity preference in the novel object preference test. Moreover, Faillace et al. [49] investigated if NIC tartrate salt (15 mg/L for 10 min) affects the innate preference of zebrafish for novel and familiar objects after short and long retention intervals. They showed that NIC significantly enhanced or changed the short term innate preference for the novel object [49].

The cognitive properties of COT and 6HLN were previously demonstrated in studies performed on rodent models of AD. Echeverria et al. [35] showed that COT administration to transgenic mice (Tg6799, bearing five familial AD mutations), before and after AD development, improved the working and reference memory and stimulated the protein kinase B (Akt) pathway activation and glycogen synthase kinase 3β (GSK3β) inhibition in the hippocampus and cortex, supporting thus the processes that underlie memory and learning such as neuronal survival and synaptic plasticity. In a double dose and a more advanced stage of the disease, COT mitigated the AD-like pathology in Tg6799 mice by improving the spatial and working memory performances [34]. The treatment also decreased the Aβ load and increased the Akt and postsynaptic density protein 95 (PSD95) expression in the hippocampus and the entorhinal cortex of the mice, supporting the synaptic plasticity [34]. Recently, we demonstrated that both COT and 6HLN ameliorated the cognitive deficits in a rat model of AD induced by intracerebroventricular (i.c.v.) infusion of Aβ_25–35_ peptide, and this effect might be related to the binding of α7 and α4β2 nAChRs [33]. Our group also evaluated the effects of chronic administration of 6HLN on spatial memory in normal Wistar rats using specific hippocampus-dependent tasks, such as Y-maze or radial arm maze [89]. Spatial memory, especially the short-term and working memory, was improved by 6HLN treatment without affecting the long-term memory [89]. Moreover, 6HLN was also found to ameliorate the cognitive deficits in SCOP- and CHL-induced rat models of AD [31,32].

Consistent with these studies, our data demonstrate that COT and 6HLN exhibit a promnesic effect in the SCOP-induced zebrafish model of AD.

### 3.3. The Effects of Cotinine and 6-Hydroxy-L-Nicotine on Acetylcholinesterase Activity

**Figure 6:**
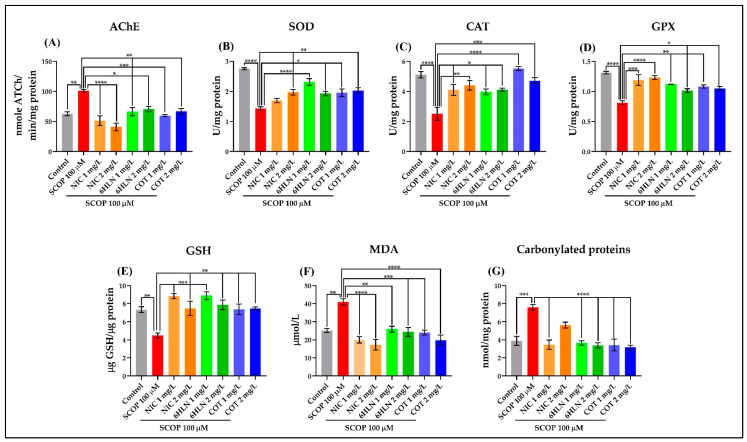
The effects of nicotine (NIC), 6-hydroxy-L-nicotine (6HLN), and cotinine (COT) (1 and 2 mg/L) administration in scopolamine (SCOP)-treated zebrafish on acetylcholine esterase (AChE)- (**A**), superoxide dismutase (SOD)- (**B**), catalase (CAT)- (**C**) and glutathione peroxidase (GPX)- (**D**) specific activities and the content of glutathione (GSH) (**E**), malondialdehyde (MDA) (**F**) and carbonylated proteins (**G**). The values are expressed as means ± S.E.M. (*n* = 3). ANOVA analysis revealed overall significant differences between the experimental groups for (**A**) F(7,16) = 11.5, *p* < 0.0001, (**B**) F(7,16) = 10.15, *p* < 0.0001, (**C**) F(7,16) = 11.31, *p* < 0.0001, (**D**) F(7,16) = 14.22, *p* < 0.0001, (**E**) F(7,16) = 8.541, *p* < 0.001, (**F**) F(7,16) = 12.39, *p* < 0.0001 and (**G**) F(7,16) = 14.53, *p* < 0.0001. For Tukey *posthoc* analyses—**** *p* < 0.0001, *** *p* < 0.001, ** *p* < 0.01 and * *p* < 0.05.

According to the cholinergic hypothesis, the AChE enzyme hydrolyzes the neurotransmitter ACh and breakdown it into choline and acetate ions, creating thus a deficiency of ACh in the synaptic cleft leading to the termination of synaptic transmission in the brain [90]. Hence, a potential therapeutic strategy is to increase the ACh levels by using AChE inhibitors that limit the ACh degradation [91]. However, the current AChE inhibitors used in AD therapy have shown various dose-associated side-effects [90,91]. In this experiment, we evaluated whether COT and 6HLN have any effect on AChE biological activity in the brain of SCOP-treated zebrafish. As depicted in Figure 6A, the acute exposure of zebrafish to SCOP (100 µM) caused a significant increase (*p* < 0.01) of AChE-specific activity compared to the control animals. Both doses of 1 and 2 mg/L of COT and 6HLN counteracted the SCOP effect, significantly reducing the AChE-specific activity in the brain of SCOP-treated zebrafish to a level close to control (Figure 6A). In a previous study, Zanandrea et al. [46] showed that SCOP administration (100 µM for 1 h) to zebrafish did not alter the specific activity of AChE compared to control fish. In contrast, several other reports have demonstrated that SCOP (100 µM for 30 min) increased the activity of this enzyme in the zebrafish brains [36,37,38,92,93]. Ziani et al. [41] showed that NIC (1 mg/L for 3 min) increases the AChE activity when it is co-administered with a compound that provokes a fear response in zebrafish but not when NIC is administered alone. Moreover, the nicotinic derivatives, COT, and 6HLN, reduce the AChE-specific activity in the hippocampus of the rats treated i.c.v. with Aβ_25–35_ peptide [33]. Coherent with these studies, our data indicate an anti-AChE profile of COT and 6HLN in the zebrafish model of AD induced by SCOP.

### 3.4. The Effects of Cotinine and 6-Hydroxy-L-Nicotine on Oxidative Stress

The oxidative stress is a pathophysiologic imbalance between oxidants (e.g. reactive oxygen species, ROS) and antioxidant defenses in favor of the former and is a contributing factor to AD pathogenesis and progression [94,95]. Here, we assessed the effects of COT and 6HLN on SCOP-induced oxidative stress in the zebrafish brain by measuring the SOD, CAT, GPX enzymes specific activities along with the content of GSH, MDA, and carbonylated proteins. The acute exposure of zebrafish to SCOP (100 µM) significantly (*p* < 0.0001) reduced the specific activities of the enzymatic scavengers of ROS, such as SOD (Figure 6B), CAT (Figure 6C), or GPX (Figure 6D) compared to control group. Also, the SCOP treatment significantly decreased the GSH content (Figure 6E) and increased the brain levels of MDA (Figure 6F), the main end product of lipid peroxidation, and carbonylated proteins (Figure 6G), a marker of protein oxidation. Alternatively, the acute administration of both doses of 1 and 2 mg/L of 6HLN and COT blocked the oxidant properties of SCOP in the zebrafish brain. Therefore, the nicotinic derivatives significantly increased the SOD (Figure 6B), CAT (Figure 6C), and GPX (Figure 6D) enzymes specific activities and the content of GSH (Figure 6E) and significantly reduced the levels of MDA (Figure 6F) and carbonylated proteins (Figure 5G) compared to the group treated with SCOP alone.

The biochemical results obtained in this study indicated that COT and 6HLN exhibit an antioxidant profile in the SCOP-induced zebrafish model of AD. Several reports showed that SCOP treatment induces oxidative stress in the brain of zebrafish [36,37,38,92,93] or rodents [64,96,97,98,99]. In a cell line derived from *Danio rerio* gill tissue, NIC treatment (3–7 mg/L for 12 h) has been shown to induce intracellular ROS generation in a dose-dependent manner [100]. Moreover, this cell line and adult zebrafish exposed to NIC (3 mg/L for 24 and 96 h, respectively) exhibited an elevation of lipid peroxidation accompanied by depletion of GSH and a reduced expression of the genes that encode manganese superoxide dismutase (*mnsod*), catalase (*cat*), glutathione peroxidase (*gpx1a*). and glutathione S-transferase (*gst*) [100]. The resulting oxidative stress followed by NIC exposure also led to an increase in the expression of apoptosis-related genes, such as *p53* and *cas3* [100]. However, these oxidant properties of NIC on zebrafish could be caused by the use of high doses and long times of exposure. According to Guan et al. [17], high doses of NIC may cause neurotoxicity and stimulate oxidative stress, while the reasonable low doses may act as an antioxidant and play a pivotal role in neuroprotection.

So far, the impact of COT and 6HLN on oxidative stress was studied in vitro and in vivo using different rodent models of AD. Srivastava et al. [101] showed that COT inhibits the generation of oxygen free radicals by neutrophils in healthy smokers and non-smokers. Moreover, COT was found to increase the hydroxyl radicals production during the autoxidation of 6-hydroxydopamine (6-OHDA), a neurotoxin widely used for studying Parkinson’s disease pathogenesis [102]. A similar effect of COT was also noticed on hydroxyl radicals generation by the Fenton reaction. This reaction was completely prevented by the preincubation of Fe^2+^ with COT [102]. In the rat brain mitochondria preparations, COT reduced the MDA production induced by the 6-OHDA autoxidation [102]. Contradictory results were obtained by de Aguiar et al. [103], which indicated that COT treatment increases both lipid peroxidation and antioxidant capacity in the rat hippocampus. However, the increased oxidative stress depends upon the dose used and is not producing memory impairments [103]. Recently, we demonstrated that COT and 6HLN reduce the Aβ_25–35_-induced oxidative stress in the rat hippocampus by stimulating the SOD, CAT, and GPX antioxidant enzyme activities, increasing the GSH content, and reducing the levels of MDA and carbonylated proteins [33]. A previous study revealed that chronic 6HLN administration improves the oxidative status by increasing the SOD- and GPX-specific activities and reducing the MDA content in the temporal cortex of normal Wistar rats [89]. An antioxidant effect was also identified in the hippocampus of SCOP- and CHL-induced rat models of AD, where the 6HLN treatment restored the SOD, CAT, and GPX enzyme-specific activities, increased the GSH content, and reduced the MDA level [31,32]. Additionally, the quantitative structure-analysis relationship (QSAR) equation and the ferric reducing ability of plasma (FRAP) test suggested that 6HLN might be a better antioxidant than NIC due to the extra hydroxyl group in the 6HLN molecule. [104,105]. Taken together, these findings indicate that COT and 6HLN possess antioxidant properties in the zebrafish model of AD.

### 3.5. The Effects of Cotinine and 6-Hydroxy-L-Nicotine on Gene Expression

#### 3.5.1. Brain-Derived Neurotrophic Factor (BDNF) Expression

**Figure 7:**
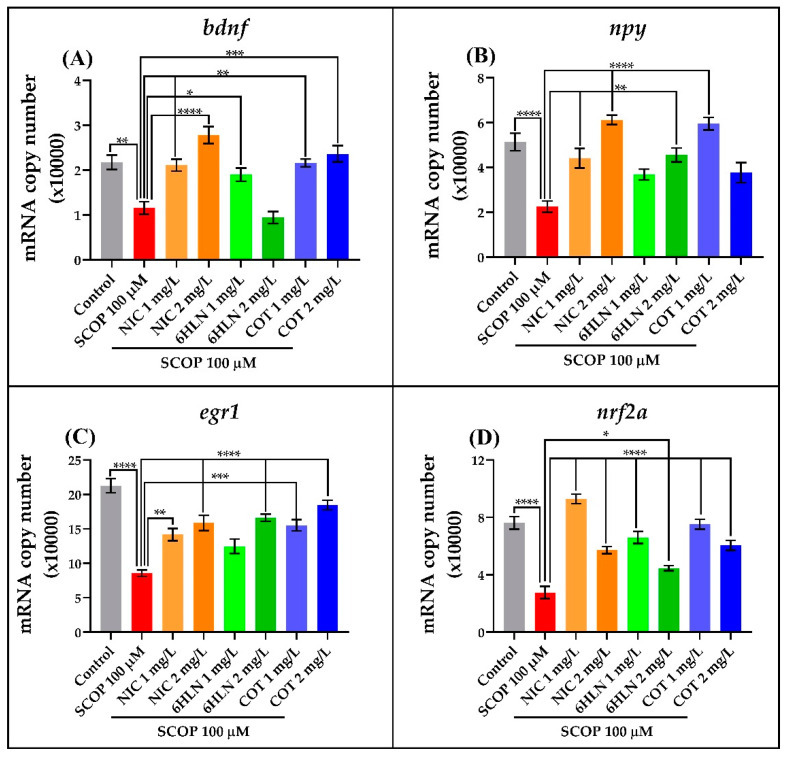
Effects of nicotine (NIC), cotinine (COT), and 6-hydroxy-L-nicotine (6HLN) (1 and 2 mg/L) administration on brain-derived neurotrophic factor (*bdnf*) (**A**), neuropeptide Y (*npy*) (**B**), early growth response protein 1 (*egr1*) (**C**), and nuclear factor erythroid 2-related factor 2 (*nrf2a*) (**D**) gene expression. The values are expressed as means ± S.E.M. (*n* = 4). ANOVA analysis identified overall significant differences between groups for (**A**) F(7,24) = 16.65, *p* < 0.0001, (**B**) F(7,24) = 14.79, *p* < 0.0001, (**C**) F(7,24) = 19.87, *p* < 0.0001 and (**D**) F(7,24) = 32.96, *p* < 0.0001. For Tukey *posthoc* analyses—**** *p* < 0.0001, *** *p* < 0.001, ** *p* < 0.01 and * *p* < 0.05.

Brain-derived neurotrophic factor (BDNF) is an important growth factor that belongs to the neurotrophin family and regulates neuronal survival, differentiation and plasticity by activating the tyrosine receptor kinase B (TrkB) and p75, the low-affinity neurotrophin receptor [106]. A reduced expression of BDNF (both mRNA and protein) was observed in post-mortem brain samples collected from AD patients, and it was reported from an early stage of the disease, such as mild cognitive impairment (MCI) [106]. Here, we evaluated the effects of COT and 6HLN on *bdnf* gene expression in the brain of SCOP-treated zebrafish. According to Figure 7A, acute administration of SCOP significantly reduced (*p* < 0.01) the mRNA copy number of the *bdnf* gene in the zebrafish brain compared to the control group. Only the 1 mg/L dose of 6HLN ameliorated the negative effect of SCOP on *bdnf* gene expression by significantly increase (*p* < 0.05, Figure 7A) the mRNA copy number. Additionally, both doses of COT were found to be effective in normalizing the *bdnf* gene expression in the brain of SCOP-treated zebrafish (*p* < 0.01 and *p* < 0.001 for 1 mg/L and 2 mg/L doses, respectively, Figure 7A). According to Chen et al. [107], SCOP treatment reduced the BDNF and TrkB immunoreactivity in the mouse dentate gyrus. Similar results were also obtained by Ionita et al. [64], which showed that SCOP intraperitoneal administration to rats induces a reduction of *bdnf* gene expression in the hippocampus. The majority of the studies conducted in humans indicated increased peripheral BDNF levels as a result of NIC exposure [108]. Such increases in BDNF levels were also observed in different animal models of AD treated with NIC [33,109]. Similar to NIC, the nicotinic derivatives, COT and 6HLN, increased the *bdnf* gene expression in the hippocampus of the rat model of AD induced by i.c.v. infusion of Aβ_25–35_ peptide [33]. Recently, Sadigh-Eteghad et al. [110] showed that chronic COT treatment restores the normal BDNF level and increases the α7 nAChRs expression in the hippocampus of aged mice. They also indicated that this effect was α7 nAChRs-dependent, as methyllycaconitine, an antagonist of these receptors, blocked the impact of COT and decreased the BDNF level and α7 nAChRs expression [110]. These findings were in line with ours, as we previously showed, using in silico tools, that both COT and 6HLN might bind with similar or higher affinity than NIC to α7 nAChRs and positively modulate their function [33]. Moreover, the treatment of neuronal cells with COT was found to be neuroprotective against Aβ- [111,112] and 6-OHDA-induced [113] cytotoxicity. These results indicate that COT and 6HLN might be involved in neuroprotection in SCOP-treated zebrafish by upregulating the *bdnf* gene expression following α7 nAChRs modulation.

#### 3.5.2. Neuropeptide Y (NPY) Expression

One of the most abundant neuropeptides in the brain is the neuropeptide Y (NPY), a 36-amino acid peptide involved in many physiological functions, including cognition [114]. Low levels of NPY have been observed in the brain, plasma, and cerebrospinal fluid of patients with AD [114,115], and NPY-expressing neurons were reported to be strongly affected in the cortex and hippocampus of transgenic AD mice [114,116]. The expression level of the *npy* gene was assessed in the brains of zebrafish co-treated with SCOP and the nicotinic derivatives, COT and 6HLN. As depicted in Figure 7B, the mRNA copy number of *npy* gene was strongly reduced (*p* < 0.0001) in the brain of the fish treated only with SCOP (100 µM) compared to the control group. The acute administration of 2 mg/L dose of 6HLN to SCOP-treated zebrafish significantly increased (*p* < 0.01, Figure 7B) the *npy* expression, although a slight increase (not significant) of mRNA copy number was also noticed for 1 mg/L dose. In contrast, COT was found to be more effective at the 1 mg/L dose, normalizing the mRNA copy number of *npy* gene in the brain of the zebrafish treated with SCOP (100 µM). An early study performed on mice showed that exogenous NPY alleviates amnesia caused by SCOP [117]. According to Li et al. [118], NIC administration upregulated the NPY expression (both mRNA and peptide level) in the rat hypothalamus. Rangani et al. [119] showed that NIC and two NPY Y1 receptor agonists, NPY and [Leu^31^, Pro^34^]-NPY improve learning and memory in a colchicine-induced rat model of AD. These agonists potentiated the NIC effects, while BIBP3226, an NPY Y1 antagonist, attenuated them [119]. Moreover, NIC restored the NPY-immunoreactivity in the nucleus accumbens shell, central nucleus amygdala, dentate gyrus, and hypothalamic arcuate, which was decreased by the AD-like condition [119]. These findings suggested that NPY interacts with the endogenous cholinergic system via NPY Y1 receptors [119]. The hypothalamic NPY neurons express α7 nAChRs and are stimulated by the NIC. The excitation of these neurons is partially mediated via α7 nAChRs, as methyllycaconitine reduces NIC action [120]. Thereby, due to structural similarities to NIC, the nicotinic derivatives, COT, and 6HLN might bind to α7 nAChRs [33] and modulate their function leading to a potential increase of NPY expression.

#### 3.5.3. Early Growth Response Protein 1 (Egr1) Expression

Early growth response protein 1 (Egr1) is a transcription factor that plays a pivotal role in processes underlying neuronal activity, from neurotransmission and synaptic plasticity to higher-order processes such as memory and learning. Its neuronal expression is induced by activity-dependent synaptic plasticity upon learning [121]. It was shown that Egr1 is upregulated in humans during the asymptomatic stages of AD but not in those symptomatic [121,122]. We evaluated the expression level of the *egr1* gene in the brain of the zebrafish co-treated with SCOP and nicotinic derivatives. Figure 7C shows that the treatment with SCOP (100 µM) determined a significant decrease (*p* < 0.0001) of the mRNA copy number of *egr1* gene in the zebrafish brain compared with control animals. Regarding the nicotinic derivatives, Tukey’s multiple comparison tests revealed that both 6HLN and COT ameliorated the negative effect of SCOP on *egr1* expression. 6HLN was found to be effective only when the higher dose was used (*p* < 0.0001 for 2 mg/L dose), although a slight increase was also noticed for 1 mg/L dose (Figure 7C). COT increased the mRNA copy number in the brain of SCOP-treated zebrafish in a dose-dependent manner (*p* < 0.001 for 1 and *p* < 0.0001 for 2 mg/L dose respectively). SCOP decreased memory consolidation and induced downregulation of several immediate early genes, including *egr1* [123]. Similar results were also obtained by Lu et al. [99], which demonstrated that SCOP significantly reduces the Egr1 protein level in the brain of the mice. Dunckley and Lucas [124] showed that acute NIC administration increases *egr1* expression in the neuronal SH-SY5Y cell line. Moreover, the microarray assay performed by Belluardo et al. [125] revealed that acute intermittent NIC treatment increased both *egr1* and *egr2* mRNA levels in various regions of the rat brain. Xue et al. [126] suggested that low doses of NIC can activate the MAPK/ERK/EGR1 signaling pathway partially through α7 nAChRs and ameliorate Aβ_25–35_-induced neurotoxicity. These results suggest that COT and 6HLN might enhance cognitive functions, such as memory and learning, by upregulating the *egr1* gene expression.

#### 3.5.4. Nuclear Factor Erythroid 2-Related Factor 2 (Nrf2a) Expression

Nuclear factor erythroid 2-related factor 2 (Nrf2) is a transcription factor that regulates several antioxidant and cytoprotective genes to protect against ROS cytotoxicity [127]. Taking into consideration that oxidative stress is involved in AD, the release, stabilization, and nuclear translocation of Nrf2 represent a cellular mechanism for suppressing this phenomenon [128]. It has been reported that nuclear Nrf2 expression is reduced in the brains of AD patients, despite the presence of oxidative stress [127,128]. The *nrf2a* gene expression level was assessed in the brain of the zebrafish co-treated with SCOP and nicotinic derivatives. According to Figure 7D, acute SCOP exposure strongly reduced (*p* < 0.0001) the mRNA copy number of the *nrf2a* gene in the zebrafish brain compared to the control group. Both doses of 6HLN, especially the 1 mg/L dose, significantly increased (*p* < 0.0001 for 1 mg/L and *p* < 0.05 for2 mg/L respectively, Figure 7D) the mRNA copy number when it was co-administered with SCOP. Besides, both doses of COT, but especially 1 mg/L, remarkably enhanced (*p* < 0.0001 for 1 mg/L, Figure 7D) the *nrf2a* expression at mRNA level in the brain of SCOP-treated zebrafish compared to the negative control. Wan et al. [129] showed that SCOP administration reduced the Nrf2 protein expression in the hippocampus and cortex of the mice and induced oxidative stress by increasing the MDA level and lowering the antioxidant capacity in these brain regions. In C6 glioma cells, SCOP decreased Nrf2 protein expression in a dose-dependent manner and significantly decreased SOD-, CAT-, and GPX-specific activities [130]. Kasnak et al. [131] showed that NIC treatment increased the Nfe2l2/Nrf2 protein expression in human gingival keratinocytes. Moreover, Lee et al. [132] demonstrated that human periodontal ligament cells exposed to NIC expressed high Nrf2 protein levels in nuclear fractions. These results suggest that the antioxidant properties of COT and 6HLN might be attributed to the upregulation of *nrf2a* gene expression by these compounds.

### 3.6. Pearson Correlations between Behavioral, Biochemical, and Genetic Parameters

Pearson’s correlation coefficient (r) was calculated to correlate several behavioral or biochemical parameters with MDA, the end product of lipid peroxidation, or *nrf2a*, *egr1*, and *npy* gene expression levels.

**Figure 8:**
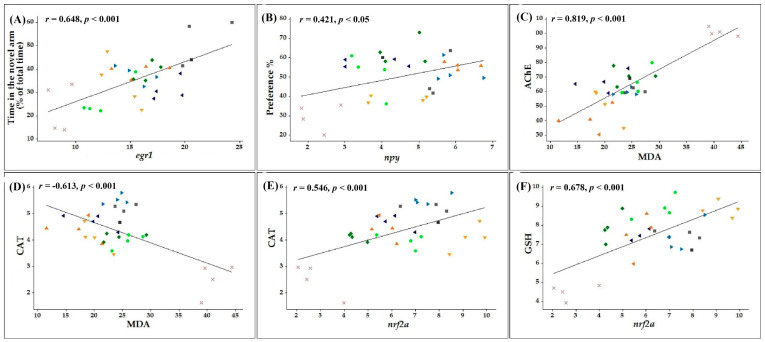
Pearson’s correlation coefficient between behavioral or biochemical parameters and malondialdehyde (MDA) or markers of gene expression (*n* = 10 animals per group): (**A**) Time in the novel arm (% of total time) vs. early growth response protein 1 (*egr1*) (*r* = 0.648, *p* < 0.001), (**B**) Preference % vs. neuropeptide Y (*npy*) (*r* = 0.421, *p* < 0.05), (**C**) Acetylcholine esterase (AChE) vs. malondialdehyde (MDA) (*r* = 0.819, *p* < 0.001), (**D**) Catalase (CAT) vs. malondialdehyde (MDA) (*r* = −0.613, *p* < 0.001), (**E**) Catalase (CAT) vs nuclear factor erythroid 2-related factor 2 (*nrf2a*) (*r* = 0.546, *p* < 0.001) and (**F**) The content of glutathione (GSH) vs. nuclear factor erythroid 2-related factor 2 (*nrf2a*) (*r* = 0.678, *p* < 0.001) in control (■), scopolamine (SCOP) (X), nicotine (NIC) 1 mg/L + scopolamine (SCOP) (▼), nicotine (NIC) 2 mg/L + scopolamine (SCOP) (▲), 6-hydroxy-L-nicotine (6HLN) 1 mg/L + scopolamine (SCOP) (●), 6-hydroxy-L-nicotine (6HLN) 2 mg/L + scopolamine (SCOP) (♦), cotinine (COT) 1 mg/L + scopolamine (SCOP) (►) and cotinine (COT) 2 mg/L + scopolamine (SCOP) (◄). Data are expressed as follows: AChE (nmol ATCh/min/mg protein), MDA (µmol/L), CAT (U/mg protein), GSH (µg GSH/µg protein), *nrf2a* (mRNA copy number, ×10,000) and *egr1* (mRNA copy number, × 10,000).

Firstly, we identified several correlations between behavioral scores measured in memory tests and the expression levels of genes involved in cognitive functions. Our results indicate that the time spent by the zebrafish in the novel arm of the Y-maze strongly correlates with the *egr1* (*n* = 10, *r* = 0.648, *p* < 0.001, Figure 8A) and *npy* (*n* = 10, *r* = 0.524, *p* < 0.01, Supplementary Figure S2A) gene expression levels. Moreover, positive correlations were also observed between preference percentages calculated within object discrimination task and the mRNA copy number of *npy* (*n* = 10, *r* = 0.421, *p* < 0.05, Figure 8B) and *egr1* (*n* = 10, *r* = 0.557, *p* < 0.001, Supplementary Figure S2B) genes. This suggests that the increase of the memory performances in the groups co-treated with SCOP and COT or 6HLN are well correlated with the upregulation of *egr1* and *npy* gene expression.

Secondly, we identified a strong positive correlation between the specific activity of AChE and the MDA level (*n* = 10, *r* = 0.819, *p* < 0.001, Figure 8C), thus suggesting an intense activity of this enzyme in the zebrafish brain when the MDA level is high. Moreover, our data revealed high negative correlations between the components of the antioxidant defense system and the MDA level as follows: CAT vs. MDA (*n* = 10, *r* = −0.613, *p* < 0.001, Figure 8D), GPX vs. MDA (*n* = 10, r = −0.692, *p* < 0.001, Supplementary Figure S2C) and GSH vs. MDA (*n* = 10, *r* = −0.678, *p* < 0.001, Supplementary Figure S2D). These findings suggest that the stimulation of the antioxidant defense system by the nicotinic derivatives is well correlated with a low MDA level.

Finally, we calculated the linear regression between the biochemical parameters of oxidative stress and the *nrf2a* gene expression level. The CAT (*n* = 10, *r* = 0.546, *p* < 0.0001, Figure 8E) and GPX (*n* = 10, *r* = 0.697, *p* < 0.001, Supplementary Figure S2E) specific activities and the content of GSH (*n* = 10, *r* = 0.678, *p* < 0.001, Figure 8F) were positively correlated with the mRNA copy number of *nrf2a* gene. However, that was not the case for the MDA level (*n* = 10, *r* = −0.577, *p* < 0.001, Supplementary Figure S2F), which was negatively correlated with the *nrf2a* gene expression level. Thereby, a reduction of SCOP - induce brain oxidative stress by COT and 6HLN is associated with an upregulation of *nrf2a* gene expression.

According to correlations performed by Hritcu et al. [89], the antioxidant properties of 6HLN might be associated with the cognitive enhancements observed in normal rats. Additionally, it has been suggested that the involvement of 6HLN in neuroprotection against SCOP-induced oxidative stress is associated with increased behavioral scores in memory tasks and increased antioxidant defense along with a low level of MDA [32]. Recently, we have shown that the antioxidant properties of 6HLN are well correlated with memory improvement and anti-AChE abilities in a rat model of AD induced by Aβ_25–35_ peptide [33]. In the brain of Tg6799 mice, the promnesic effects of COT were explained by COT ability to prevent Aβ aggregation and to reduce its levels [35]. Furthermore, the pro-cognitive effects of COT were also associated with an inhibition of the GSK3β enzyme in a mouse model of Fragile X syndrome [133].

## 4. Conclusions

The present study was conducted to evaluate the anxiolytic and anti-amnesic activities of nicotinic derivatives, COT and 6HLN, in a SCOP-induced zebrafish model of AD. Our data demonstrated that nicotinic derivatives administration ameliorated anxiety and cognitive deficits measured by performance in specific tasks. Besides, the treatment with nicotinic derivatives reduced the AChE activity, improved the antioxidant system, and decreased the oxidative stress (lipid and protein oxidation) in the brain of SCOP-treated zebrafish. The cognitive-enhancing abilities and antioxidant properties of COT and 6HLN might be due to the increase of *bdnf*, *npy*, *egr1*, and *nrf2a* gene expression as a response to a potential modulation of the α7 nAChRs. The results indicate that the underlying mechanism of memory improvement may involve modulations of the cholinergic system, specific gene expression, and the reduction of oxidative stress. This evidence suggests that COT and 6HLN could be considered a viable therapeutic alternative for ameliorating the AD condition.

## Figures and Tables

**Table 1:** Primers used to amplify the genes of interest in *Danio rerio.*

Gene	Product Size (bp)	Primer	Sequence	Reference Sequence
*npy*	104	Forward	5′-GAC TCT CAC AGA AGG GTA TCC-3′	NM_131074.2
		Reverse	5′-GGT TGA TGT AGT GTC TTA GTG CTG-3′	
*bdnf*	83	Forward	5′-GCT CTC TCA ATG CGC ACT AC-3′	NM_131595.2
		Reverse	5′-TGA CTG AGC GGA TCC TTT GG-3′	
*egr1*	110	Forward	5′-AGT TTG ATC ACC TTG CTG GAG-3′	NM_131248.1
		Reverse	5′-AAC GGC CTG TGT AAG ATA TGG-3′	
*nrf2a*	106	Forward	5′-ATG TCT AAA ATG CAG CCA AGC C-3′	NM_182889.1
		Reverse	5′-CGG TAG CTG AAG TCG AAC AC-3′	

## Data Availability

The data presented in this study are available on request from the corresponding author.

## Supplementary Materials

The following are available online at https://www.mdpi.com/2076-3921/10/2/212/s1. Figure S1: The effects of nicotine (NIC), 6-xydroxy-L-nicotine (6HLN) and cotinine COT (1 and 2 mg/L) administration in scopolamine (SCOP)-treated zebrafish on anxiety-like behavior evaluated within the novel tank diving test (NTT); Figure S2: Pearson’s correlation coefficient between behavioral or biochemical parameters and malondialdehyde (MDA) or markers of gene expression (*n* = 10 animals per group).
