# Supplementary material for: Anxiolytic, Promnesic, Anti-Acetylcholinesterase and Antioxidant Effects of Cotinine and 6-Hydroxy-L-Nicotine in Scopolamine-Induced Zebrafish (Danio rerio) Model of Alzheimer’s Disease
Source: Antioxidants (Basel). 2021 Feb 1;10(2):212. doi: 10.3390/antiox10020212 (PMC7912787; doi:10.3390/antiox10020212)
Supplement: Supplementary file 1 [file antioxidants-10-00212-s001.pdf]

*Supplementary material*

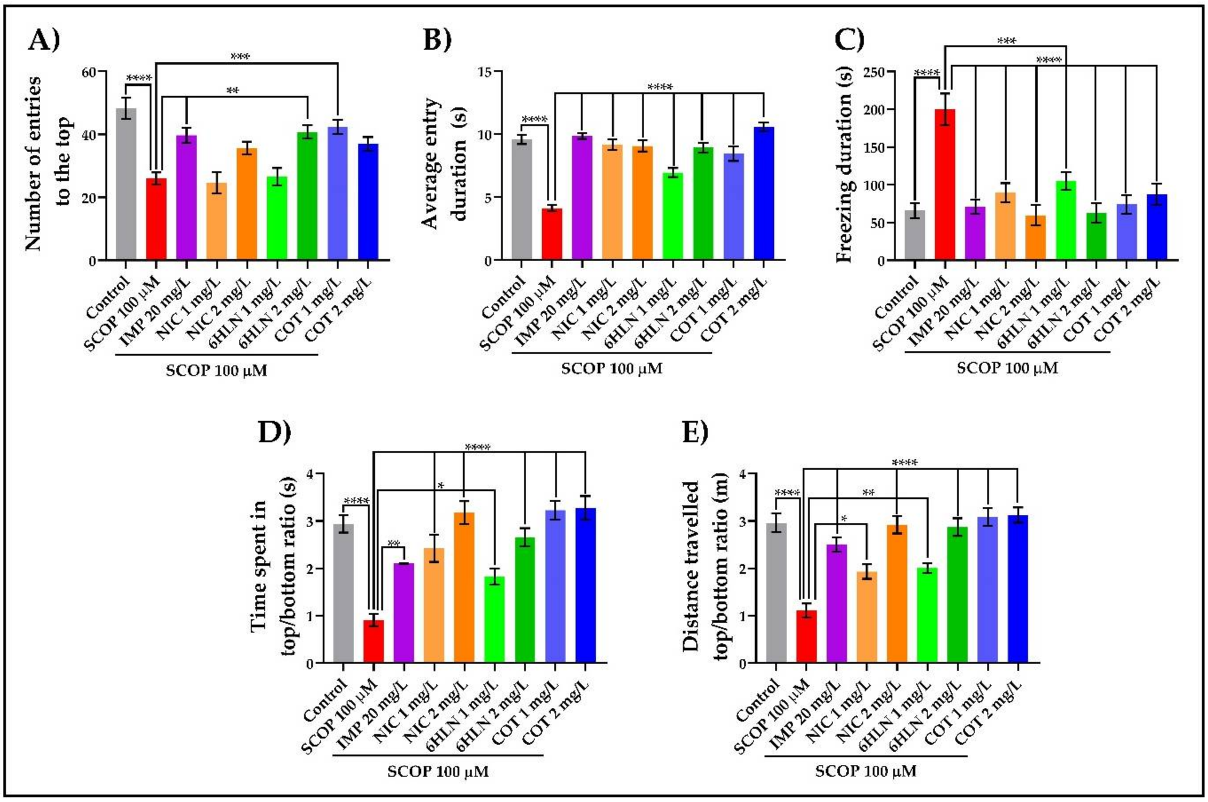


**Figure S1**. The effects of NIC, 6HLN and COT (1 and 2 mg/L) administration in SCOP-treated zebrafish on anxiety-like behavior evaluated within NTT. The number of entries to the top (**A**), average entry duration (**B**) freezing duration (**C**), time spent in top/bottom ratio (**D**) and distance traveled top/bottom ratio (**E**) were the additional parameters for measuring the anxiety like behavior. The values are expressed as means ± S.E.M. (n=10). ANOVA analysis identified overall significant differences between the experimental groups for (**A**) $F(8,81)=10.41$, $p<0.0001$, (**B**) $F(8,81)=25.10$, $p<0.0001$, (**C**) $F(8,81)=10.68$, $p<0.0001$, (**D**) $F(8,81)=15.4$, $p<0.0001$ and (**E**) $F(8,81)=17.11$, $p<0.0001$. For Tukey *post hoc* analyses – **** $p<0.0001$, *** $p<0.001$, ** $p<0.01$ and * $p<0.05$.

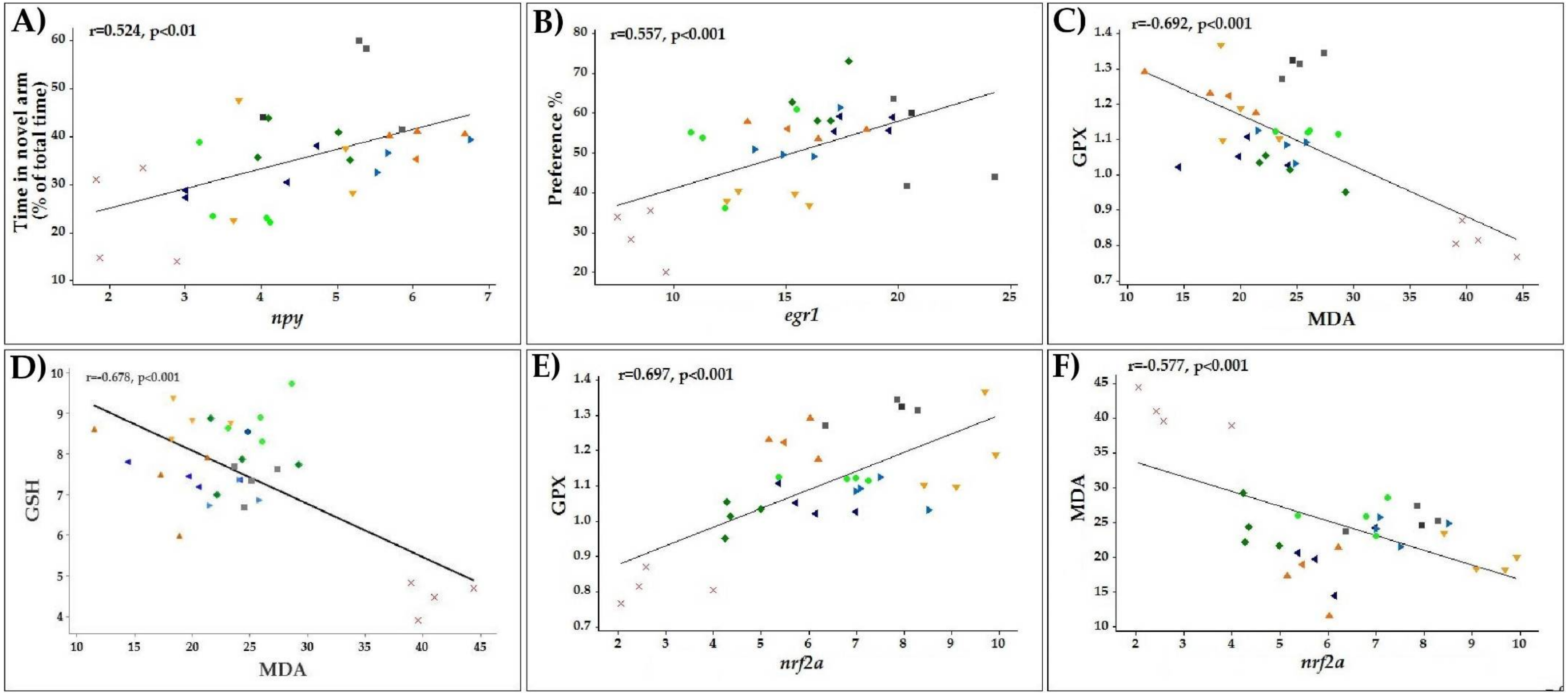


**Figure S2**. Pearson's correlation coefficient between behavioral or biochemical parameters and MDA or markers of gene expression (n=10 animals per group): (**A**) Time in the novel arm (% of total time) vs. *npy* (r=0.524, p<0.01), (**B**) Preference % vs. *egr1* (r=0.557, p<0.001), (**C**) GPX vs. MDA (r=-0.692, p<0.001), (**D**) GSH vs. MDA (r=-0.678, p<0.001), (**E**) GPX vs *nrf2a* (r=0.697, p<0.001) and (**F**) MDA vs. *nrf2a* (r=0.678, p<0.001) in control ( ), SCOP (x), NIC 1 mg/L + SCOP (▼), NIC 2 mg/L + SCOP (▲), 6HLN 1/mg/L + SCOP (●), 6HLN 2 mg/L + SCOP (◆), COT 1 mg/L + SCOP (▶) and COT 2 mg/L + SCOP (◀). Data are expressed as follow: MDA (μmol/L), GPX (U/mg protein), GSH (μg GSH/μg protein), *nrf2a* (mRNA copy number, x10000), *egr1* (mRNA copy number, x10000) and *npy* (mRNA copy number, x10000).
